# A Functional Near-Infrared Spectroscopy Study of State Anxiety and Auditory Working Memory Load

**DOI:** 10.3389/fnhum.2018.00313

**Published:** 2018-08-07

**Authors:** Yi-Li Tseng, Chia-Feng Lu, Shih-Min Wu, Sotaro Shimada, Ting Huang, Guan-Yi Lu

**Affiliations:** ^1^Department of Electrical Engineering, Fu Jen Catholic University, New Taipei City, Taiwan; ^2^Department of Biomedical Imaging and Radiological Sciences, National Yang-Ming University, Taipei, Taiwan; ^3^Department of Electronics and Bioinformatics, School of Science and Technology, Meiji University, Tokyo, Japan

**Keywords:** functional near-infrared spectroscopy (fNIRS), auditory working memory, memory load, state anxiety, prefrontal cortex (PFC)

## Abstract

Cognitive studies have suggested that anxiety is correlated with cognitive performance. Previous research has focused on the relationship between anxiety level and the perceptual load within the frontal region, such as the dorsolateral prefrontal and anterior cingulate cortices. High-anxious individuals are predicted to have worse performance on cognitively-demanding tasks requiring efficient cognitive processing. A few functional magnetic resonance imaging studies have specifically discussed the performance and brain activity involving working memory for high-anxious individuals. This topic has been further explored with electroencephalography, although these studies have mostly provided results involving visual face-related stimuli. In this study, we used auditory stimulation to manipulate the working memory load and attempted to interpret the deficiency of cognitive function in high-anxious participants or patients using functional near infrared spectroscopy (fNIRS). The fNIRS signals of 30 participants were measured while they were performing an auditory working memory task. For the auditory *n*-back task, there were three experimental conditions, including two *n*-back task conditions of stimuli memorization with different memory load and a condition of passive listening to the stimuli. Hemodynamic responses from frontal brain regions were recorded using a wireless fNIRS device. Brain activation from the ventrolateral and orbital prefrontal cortex were measured with signals filtered and artifacts removed. The fNIRS signals were then standardized with statistical testing and group analysis was performed. The results revealed that there were significantly stronger hemodynamic responses in the right ventrolateral and orbital prefrontal cortex when subjects were attending to the auditory working memory task with higher load. Furthermore, the right lateralization of the prefrontal cortex was negatively correlated with the level of state anxiety. This study revealed the possibility of incorporating fNIRS signals as an index to evaluate cognitive performance and mood states given its flexibility regarding portable applications compared to other neuroimaging techniques.

## Introduction

### Anxiety disorder and cognitive function

Neurophysiological studies have suggested that anxiety is highly correlated with cognitive performance (Eysenck et al., 2007; Osinsky et al., 2012). The level of anxiety in these studies was assessed by trait anxiety as measured by, for instance, the State-Trait Anxiety Inventory (STAI) (Spielberger et al., 1970). State anxiety is conceptualized as a transient emotional state during which an individual is unable to respond to events that are threatening an existing goal (Eysenck and Calvo, 1992; Eysenck et al., 2007; Power and Dalgleish, 2015), whereas trait anxiety is a relatively stable personality characteristic (Horwitz, 2001; Schmukle and Egloff, 2004). Investigations have focused on prefrontal mechanisms and top-down selective attention to threatening events (Bishop et al., 2004; Ohman, 2005). In highly anxious subjects, attentional control is more likely to be influenced by emotional stimuli, particularly concerning tasks engaging conflict stimuli with low working memory load (Osinsky et al., 2012; Pacheco-Unguetti et al., 2012; Vytal et al., 2012). Moreover, cognitive processes are also affected in high-anxious subjects in the absence of threat-related stimuli (Basten et al., 2012). These studies have mainly focused on the relationship between the anxiety level and working memory or perceptual load in the frontal region, especially in the prefrontal cortex (Bishop, 2009; Basten et al., 2012).

These recent studies have claimed that there is interference of trait anxiety in inhibition and attentional shift in working memory. The ease of “overload” in anxious subjects resulting in diluting cognitive resources particularly affects the processes associated with inhibition and attentional shift (Berggren and Derakshan, 2013), and can be observed when there is lower cognitive load (Berggren et al., 2013). There is no doubt that high-anxious subjects are predicted to have worse performance on cognitively demanding tasks requiring more efficient cognitive processes, and the examination of compensatory strategy will be the future direction of research (Ansari and Derakshan, 2011a; Berggren and Derakshan, 2013).

### Neuroimaging evidence for anxiety and cognitive impairment

Recent functional magnetic resonance imaging (fMRI) studies have reported varying brain activities in frontal regions for subjects with distinct anxiety levels (Bishop et al., 2004; Bishop, 2009; Basten et al., 2011, 2012). Basten et al. reported that there is stronger task-relevant activation in high-anxious subjects in the dorsal lateral prefrontal cortex (DLPFC) and left inferior frontal sulcus (Basten et al., 2011, 2012). Stronger deactivation was found in the rostral-ventral anterior cingulate cortex (ACC), which is one of the main regions in the default-mode network (Basten et al., 2012). Higher levels of anxiety were associated with stronger task-related activation in the ACC but with reduced functional connectivity between the ACC and lateral prefrontal cortex (LPFC) (Comte et al., 2015). In addition, there is reduced functional connectivity of the DLPFC with posterior lateral frontal regions, the dorsal ACC, and a word-sensitive area in the left fusiform gyrus (Basten et al., 2011), whereas stronger connectivity is found for the right DLPFC with the ventrolateral PFC in high-anxious subjects (Basten et al., 2012). Some of these recent studies have found stronger or compensatory activation in certain brain regions for high-anxious participants with comparable levels of performance accuracy (Ansari and Derakshan, 2011a; Basten et al., 2011). However, other studies have found weaker activation in high-anxious subjects with slower performance (Bishop, 2009; Ansari and Derakshan, 2011b). Altered neurophysiological signals including electroencephalography (EEG) and magnetoencephalography (MEG) have also been reported for subjects with different mental illness and anxiety levels. High-anxious subjects may show atypical event-related potential/field (ERP/ERF) and event-related spectral perturbations (ERSPs). Recent EEG studies have shown that there is altered activation in subjects showing high anxiety in cognitive tasks such as attention (Osinsky et al., 2012), working memory, and emotional stimuli (Dennis and Hajcak, 2009; MacNamara and Hajcak, 2009, 2010; Ansari and Derakshan, 2011a,b; MacNamara et al., 2011; Hajcak et al., 2013; Stout et al., 2013). Lower ERP is found in high-anxious participants especially in the frontal and central regions (Ansari and Derakshan, 2011a,b). Recently in time-frequency ERSPs studies, Cavanagh and colleagues found that frontal-midline theta signals, reflecting midcingulate cortex activity, are moderated by anxiety and may predict adaptive behavioral adjustments (Cavanagh and Shackman, 2014). These findings emphasize the importance of brain activation in anxiety regulation especially in the frontal and central regions.

Although neurophysiological techniques, such as EEG and MEG, provide good temporal resolution during the execution of tasks, these methods do not offer sufficient spatial resolution. In comparison, fMRI provides better spatial resolution without the influence of electrooculography and motion artifacts. Several critical brain regions related to anxiety and cognitive functions, including the DLPFC and ACC, are constantly reported in fMRI studies. Nevertheless, it is somewhat impossible to measure fMRI signals in a natural and real-life situation for further diagnosis or applications of mental training. This study intended to incorporate a portable functional near-infrared spectroscopy (fNIRS) system in order to provide a more flexible solution to measuring the performance on cognitive tasks from subjects with different anxiety levels. Compared with fMRI, an fNIRS system is able to record hemodynamic responses with a higher sampling rate under real-life environmental conditions.

### Functional NIRS as a highly flexible device to study mental diseases and cognitive functions

In the past decade, fNIRS has been proposed as a flexible and portable device to record brain hemodynamic responses during the performance of cognitive tasks such as learning, memory, and motor reactions (Shimada and Hiraki, 2006; Shimada and Oki, 2012; Noah et al., 2015). In several studies of memory function, the hemodynamic responses recorded by a fNIRS system from the PFC have been found to correlate highly with gray-matter fMRI activities during a working memory task (Sato et al., 2013; Noah et al., 2015). Consistent with the previous findings with fMRI, LPFC activation of the fNIRS signal is also reportedly associated with working memory in adults and even preschool children (Hoshi et al., 2003; Tsujimoto et al., 2004; Schreppel et al., 2008; Sanefuji et al., 2011). Hoshi and colleagues found that there are hemodynamic changes in the human LPFC during visual working memory tasks (Hoshi et al., 2003). Later, in 2008, Schreppel and colleagues used a 52-channel fNIRS system to claim that prefrontal activities reflect both maintenance and attentional monitoring processes during a visual *n*-back task (Schreppel et al., 2008). Sanefuji and colleagues provided further supporting evidence with preschool children and proposed that there is a correlation between memory ability and activation in the left ventrolateral prefrontal cortex (VLPFC) (Sanefuji et al., 2011). In line with previous results, Ogawa and colleagues highlighted that there is correlation between working memory performance and the neural activation measured using an fNIRS system. Participants with better working memory performance had higher levels of oxyhemoglobin activation (Ogawa et al., 2014). In addition, activation in the bilateral LPFC depends on memory load (Tsujimoto et al., 2004). This evidence suggests that fNIRS is useful and convenient for measuring working memory performance.

The prefrontal hemodynamic response recorded by an fNIRS system has also become an important marker in the study of emotion regulation and mental diseases. Aoki and colleagues demonstrated that fNIRS signals can be used to investigate neural processing during emotional control of negative mood. Correlation analysis has shown that the level of negative mood is inversely associated with PFC activity during verbal working memory tasks (Aoki et al., 2011, 2013; Sato et al., 2014). In contrast, the emotional valence of the pictures positively affected the level oxygenated hemoglobin in anterior parts of the medial prefrontal cortex (MPFC) and left inferior frontal gyrus during a facial emotional *n*-back task (Ozawa et al., 2014). Although Lai and colleagues reported that a normal intensity level of psycho-physiological stress can benefit working memory performance at high load (Lai et al., 2014), other fNIRS studies have claimed that there is altered prefrontal brain activities in patients with mental diseases and problems in emotional regulation. Ehlis and colleagues found reduced VLPFC activation in patients with attention-deficit/hyperactivity disorder (ADHD) during a visual working memory task (Ehlis et al., 2008). In contrast, patients with major depressive disorder (MDD) have shown different patterns with increased activations in the LPFC and superior temporal cortex during the two-back visual task and associated with poorer task performance than healthy controls (Pu et al., 2011). Furthermore, other studies have focused on altered prefrontal activation after drug treatment. Ramasubbu and colleagues reported that there are reduced prefrontal hemodynamic responses but improved cognitive performance after treatment with methylphenidate (Ramasubbu et al., 2012). These previous findings suggest that there are complicated interactions between emotional regulation, cognitive performance, and drug treatment.

In the past few years, the prefrontal region has been highlighted as critical in auditory connections that are involved in the processing of key pieces of information for auditory memory recognition in primates (Plakke et al., 2013; Plakke and Romanski, 2014). Although the existing literature on working memory has emphasized visual processes rather than auditory modalities, more studies have started to focus on the pathway of non-verbal or auditory memory (Huang et al., 2013; Plakke et al., 2013; Plakke and Romanski, 2014; Muñoz-López et al., 2015). It has also been reported that the frontal lobe may be involved in auditory detection, discrimination, and working memory in humans (Plakke and Romanski, 2014). In 2013, Huang et al. claimed that the subregions of the anterior DLPFC are selectively associated with auditory working memory, and areas in more inferior lateral aspects of the PFC and inferior frontal cortex are associated with auditory attention and not working memory (Huang et al., 2013). These studies emphasized the importance of the frontal cortex for information processing during auditory working memory. However, there remains discrepancy between the existing experimental designs of auditory memory and real-world conditions, as most previous studies focused on the primary auditory cortex with simple tones as stimuli (Weinberger, 2014).

In this study, we investigated whether changes in prefrontal activation correlated with working memory load and trait anxiety level. We recorded hemodynamic responses from an fNIRS system while participants were performing a musical *n*-back working memory task (Pallesen et al., 2010). Four-channel fNIRS signals were recorded by a portable system to address the following hypotheses: (1) the hemodynamic responses of the lateral PFC would increase with auditory working memory load, and (2) compared to the subjects with low anxiety level, high-anxious participants would have alterations in hemodynamic responses during auditory working memory, especially in the prefrontal regions. It is worth noting that this work was an extension from our proof-of-concept study (Wu et al., 2017) with considerable improvement in two aspects. First, this work proposed the correlation between anxiety level, cognitive load, and fNIRS responses. Second, advanced fNIRS signals processing methods were incorporated to improve the signal quality from portable fNIRS devices. To the best of our knowledge, this study is the first to explore the correlation between anxiety level and cognitive load incorporating fNIRS signals during a musical working memory task, with music chords serving as stimuli.

## Materials and methods

### Participants

Thirty volunteers without neurological disorders were recruited for the experiment. Prior to the experiment, all were asked to provide informed consent in accordance with the procedure of the human subject research ethics committee/Institutional Review Board at the Fu Jen Catholic University, Taiwan, and then completed a rating scale to estimate their familiarity to distinct types of music. To avoid introducing bias pertaining to differing musical skill levels, a music-familiarity scare was administered.

### STAI

The STAI was used for measuring the participants' anxiety level (Spielberger et al., 1970). The STAI measures the state anxiety (STAI-S) and trait anxiety levels (STAI-T). Both parts of the STAI include 20 questions each, which are scored on a 4-point Likert-type scale. Specifically, the STAI-S is based on the temporary and present emotional situation of the participants, while the STAI-T can evaluate the state of pressure or anxiety during a period of time. In this study, we used the Chinese version of the STAI (cSTAI), which is validated with its psychometric characteristics found in the study by Savostyanov et al. (2009) and Shek (1993). Scores range between 20 and 80 for both the STAI-S and STAI-T, with higher scores associated with high state or trait anxiety level.

### Experimental paradigm

The subjects participated in an auditory *n*-back working memory task. The proposed auditory *n*-back WM task was modified by the paradigm developed by Pallesen et al (Pallesen et al., 2010). The paradigm is useful in studying emotion regulation during cognitive tasks by manipulating the memory load and the emotional type of the stimuli. There were three experimental conditions, including two *n*-back task conditions of memorizing the stimuli, an easy 1-back task (1B) and a difficult 2-back task (2B), and a condition of passive listening to the stimuli (PL). The stimuli were 12 sound combinations of major, minor, and dissonant chords, each spanning three frequency levels separated by an octave. The chords were produced and presented using the Overture 4.0 software, and were programed to have the same duration (1,000 ms) and loudness level. The major chords consisted of A, C#, E, A, and C#. The minor chords consisted of of A, C, E, A, and C and are considered in music theory as imperfect consonance. The dissonant chords were composed of A, Bb, G, Ab, and C, which are considered as the most dissonant intervals. After each stimulus, participants were requested to press the left button in the 1B task when the chord matched that of the previous trial, and in the 2B task when the chord matched that presented two trials back. In all other trials and the PL condition, participants pressed the right button with their right hands.

**Figure 1:**
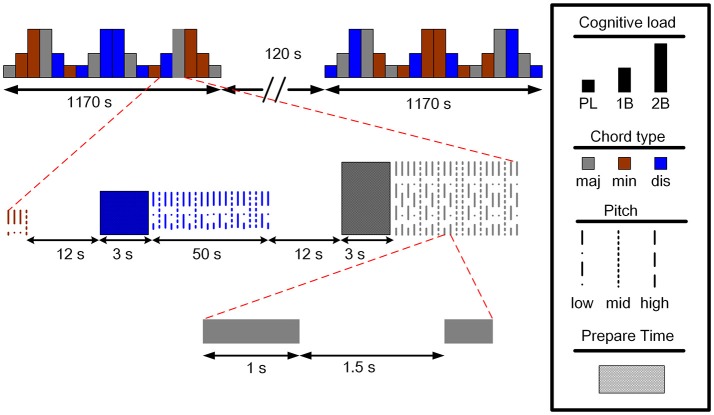
The experimental paradigm of the auditory *n*-back working memory task (maj: major, min: minor, dis: dissonant).

The experimental design is illustrated in Figure 1. There were two sessions separated by a break of 2 min in the experiment. Each session consisted of 18 blocks with different combinations of task conditions (PL, 1B, or 2B) and chord category (major, minor, or dissonant). Each type of block was presented four times during the experiment. An instruction screen was shown for 12 s between the blocks to prepare the subject for the following task. Twenty trials were presented with each trial constructed of a sound presentation (1,000 ms), followed by a brief silence (1,500 ms) before the next trial. The fNIRS signals were recorded during the two sessions, and the participants were instructed to remain calm with their eyes open while fixating on a central cross on the screen.

### fNIRS recording

**Figure 2:**
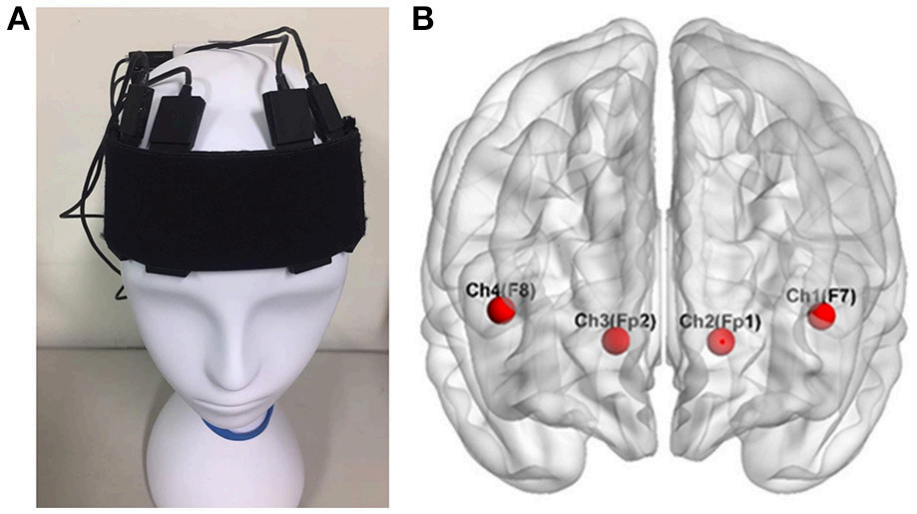
The four locations of functional near infrared spectroscopy sensors on the subject's scalp **(A)**. These electrodes corresponded to F7, F8, Fp1, and Fp2 in the 10–20 system **(B)**, located over the left and right ventrolateral prefrontal cortex and the orbital prefrontal cortex Wu et al. (2017).

The participants were recruited in the experiment with their fNIRS data recorded in a shielded room. The fNIRS signals were recorded with a sampling rate of 2 Hz using a wireless and portable system, BRAIN-NIRS Hb13 (ASTEM Co. Ltd., Kawasaki, Japan), as shown in Figure 2A. Light-emitting diodes with operating wavelengths of 770 and 830 nm are used. The concentration of oxygenated hemoglobin (oxy-Hb) and deoxygenated hemoglobin (deoxy-Hb) were recorded from four locations on the scalp. The center of the probe was placed on the frontal area (Fpz), and four sensors were set on F7, F8, Fp1, and Fp2 according to the international 10–20 system for electroencephalography, as illustrated in Figure 2B (Jasper, 1958). These four positions corresponded to the left/right VLPFC and orbital prefrontal cortex (OPFC), based on anatomical cranio-cerebral correlation studies (Okamoto et al., 2004; Sanefuji et al., 2011). The concentration change in oxy-Hb was used for further analysis, since it is more sensitive to changes in cerebral blood flow.

### fNIRS signals analysis

The HOMER2 toolbox was used for the preprocessing of oxy-Hb and deoxy-Hb (Huppert et al., 2009). Since the quality of fNIRS signals are often affected by artifacts such as respiration, eye blinking, motion, and cardiac cycle effects (Izzetoglu et al., 2005; Ayaz et al., 2010; McKendrick et al., 2014), two methods were incorporate for artifact rejection. First, the wavelet-based artifact removal algorithm (Molavi and Dumont, 2012; Brigadoi et al., 2014) was applied to the raw fNIRS signals to correct spike artifacts. Second, a band-pass filter was used to remove baseline wandering. Finally, the correlation-based signal improvement method (Cui et al., 2010) was used to eliminate the effect of motion artifacts.

The spike artifacts produced by head or body movement (Robertson et al., 2010) were removed using discrete wavelet transformation (Molavi and Dumont, 2012) modified based on the function hmrMotionCorrectWavelet.m in the HOMER2 toolbox (Huppert et al., 2009). The method calculates the difference between artifacts and fNIRS signals in terms of duration and amplitude (Brigadoi et al., 2014). The fNIRS signal, *x*(*t*), from a single channel can be expanded using the discrete wavelet transform as:

$$\begin{array}{l} x\left(t\right)=\underset{k}{\sum }v_{j_{0}k}\phi_{j_{0}k}\left(t\right)+\overset{\infty }{\underset{j=j_{0}}{\sum }}\underset{k}{\sum }w_{jk}\psi_{jk}\left(t\right), \end{array}$$

where ϕ(*t*) and ψ(*t*) are the mother wavelet and scaling functions, with *j* and *k* the dilation and translation parameters, and *j*_0_ the coarsest scale in the decomposition. The wavelet coefficients are denoted as *v*_*jk*_ and *w*_*jk*_. According to the fast wavelet transform algorithm (Mallat, 1989), the wavelet coefficients, *w*_*jk*_, are composed of the wavelet filter bank high-pass filters. The normal cumulative distribution function of *w*_*jk*_ and the variance of the distribution can be estimated. If the probability of the values is larger than *w*_*jk*_, less than a probability α, the coefficient could be classified as one produced by artifacts and should be removed (Molavi and Dumont, 2012). By reconstructing the fNIRS signals, the spike artifacts could be removed.

The fNIRS signals were then band-pass filtered (0.0015–0.02 Hz) to attenuate the high frequency noise, respiration, and cardiac cycle effects. Since the block frequency of this experimental design is close to 0.002 Hz, the low frequency of the band-pass filter was set to 0.0015 Hz to eliminate the low-frequency baseline wandering.

In the last step of signal preprocessing, the quality of the fNIRS signals was improved based on the negative correlation between oxygenated and deoxygenated hemoglobin dynamics (Cui et al., 2010). Let *x*_0_ and *y*_0_ be the true oxy- and deoxy-Hb, the recorded oxy- and deoxy-Hb, *x* and *y*, can be written as:

$$\{\begin{array}{l} x=x_{0}+\text{β}F+Noise\\ y=y_{0}+F+Noise, \end{array}$$

where *F* is the noise with identical effects on oxy- and deoxy-Hb and is subject to a constant factor β. Since the concentration of oxy-Hb and deoxy-Hb are assumed to be negative correlated, the relationship of *x*_0_ and *y*_0_ can be expressed as:

$$x_{0}=-\gamma y_{0},$$

where γ is a parameter concerning the amplitude difference between oxy and deoxy-Hb. By the assumption that (1) the amplitude of deoxy-Hb is usually lower than that of oxy-Hb, and (2) the amplitude of the noise (*F*) and true oxy-Hb are minimally correlated (Cui et al., 2010), the true amplitude of oxy- and deoxy-Hb can thus be obtained with negative correlation as:

$$\{\begin{array}{c} x_{0}=\frac{1}{2}\left(x-\beta y\right)\\ y_{0}=-\frac{1}{\beta }x_{0}. \end{array}$$

The negative correlation based signal improvement used in this study was modified from the function hmrMotionCorrectCbsi.m in the HOMER2 toolbox (Huppert et al., 2009). An index of lateralized hemodynamic responses is also proposed by subtracting the hemodynamic responses of the right VLPFC (F8) by the left VLPFC (F7). Group analysis and statistical testing were then performed after signal preprocessing.

### Statistical analysis

Statistical analysis were performed using MATLAB R2016a (The MathWorks, Inc., Natick, MA). The fNIRS signals were segmented and normalized using the average of the hemodynamic responses in 3 s before the onset of each session as a baseline. For individual analysis, the averages of the oxy-Hb change in all blocks were calculated and compared between conditions (PL, 1B, and 2B) with student's paired *t*-test. Group analyses of all participants were performed by calculating the Pearson's linear correlation coefficients between the state and trait anxiety scores with the mean oxy-Hb changes and lateralized hemodynamic responses of the data.

## Results

### Subjective rating on music familiarity, anxiety, and behavioral performance in the auditory *n*-back task

From all 30 participants recruited to the study, two were rejected due to low task accuracy (i.e., average accuracy <60%) which is close to the chance level. Consequently, a total of 28 participants (14 women) with a mean age of 21.5 ± 1.4 years were included into the data sample. Among the 28 participants, the behavior results in the auditory *n*-back task revealed that the average correctness of the 1-back task was 84.8 ± 9.6%, which is 15.5% higher than that of the 2-back task (69.3 ± 10.4%). The overall task accuracy or correctness averaged between the two conditions of the auditory working memory task was 77.0 ± 9.4%. Trait anxiety raw scores ranged from 34 to 57 with the average score of 45.8 ± 5.8, and the raw scores of state anxiety ranged from 24 to 56 with the average score of 35.9 ± 8.6. No significant correlation was found between task performance and anxiety levels. None of the participants were music experts, but most of them had systematically studied at least one instrument for no more than 5 years. In addition, most of the participants reported the habit of listening to music for at least 1 h per week. The average reaction time of the three conditions of passive listening, 1-back, and 2-back are 807 ± 257 ms, 1,149 ± 142 ms, and 1,252 ± 147 ms, respectively.

### Hemodynamic responses

**Figure 3:**
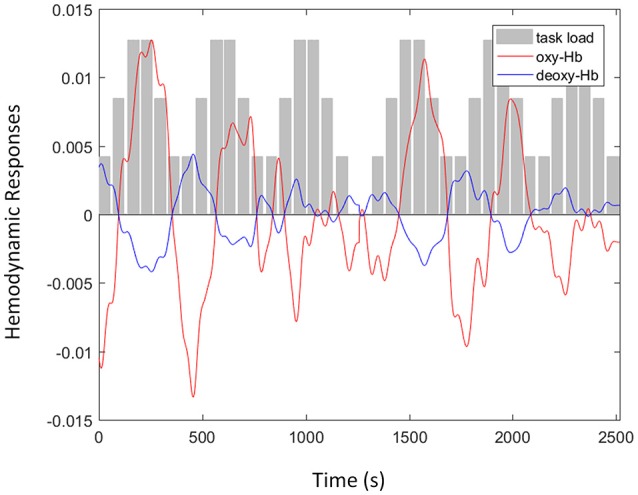
An example of functional near infrared spectroscopy signals with oxyhemoglobin (oxy-Hb) (red line) and deoxy-Hb (blue line) signals recorded at F8 from a single subject when the participant was attending to the *n*-back auditory working memory tasks including three levels of task load (gray bars): passive listening, one-back, and two-back.

The concentration of oxy- and deoxy-Hb was calculated from the fNIRS signals and is shown in Figure 3. The hemodynamic responses at F8 were recorded from a single subject when the participant was attending to the *n*-back auditory working memory task. As illustrated in Figure 3, the amplitudes of oxy-Hb signal (red line) after the removal of spike artifacts and band-pass filtering increased with the increment of task loads (gray bars). In addition, the oxy-Hb signals were negatively correlated with the amplitudes of the deoxy-Hb signal (blue line) after the removal of motion artifacts using the negative correlation-based signal improvement algorithm.

**Figure 4:**
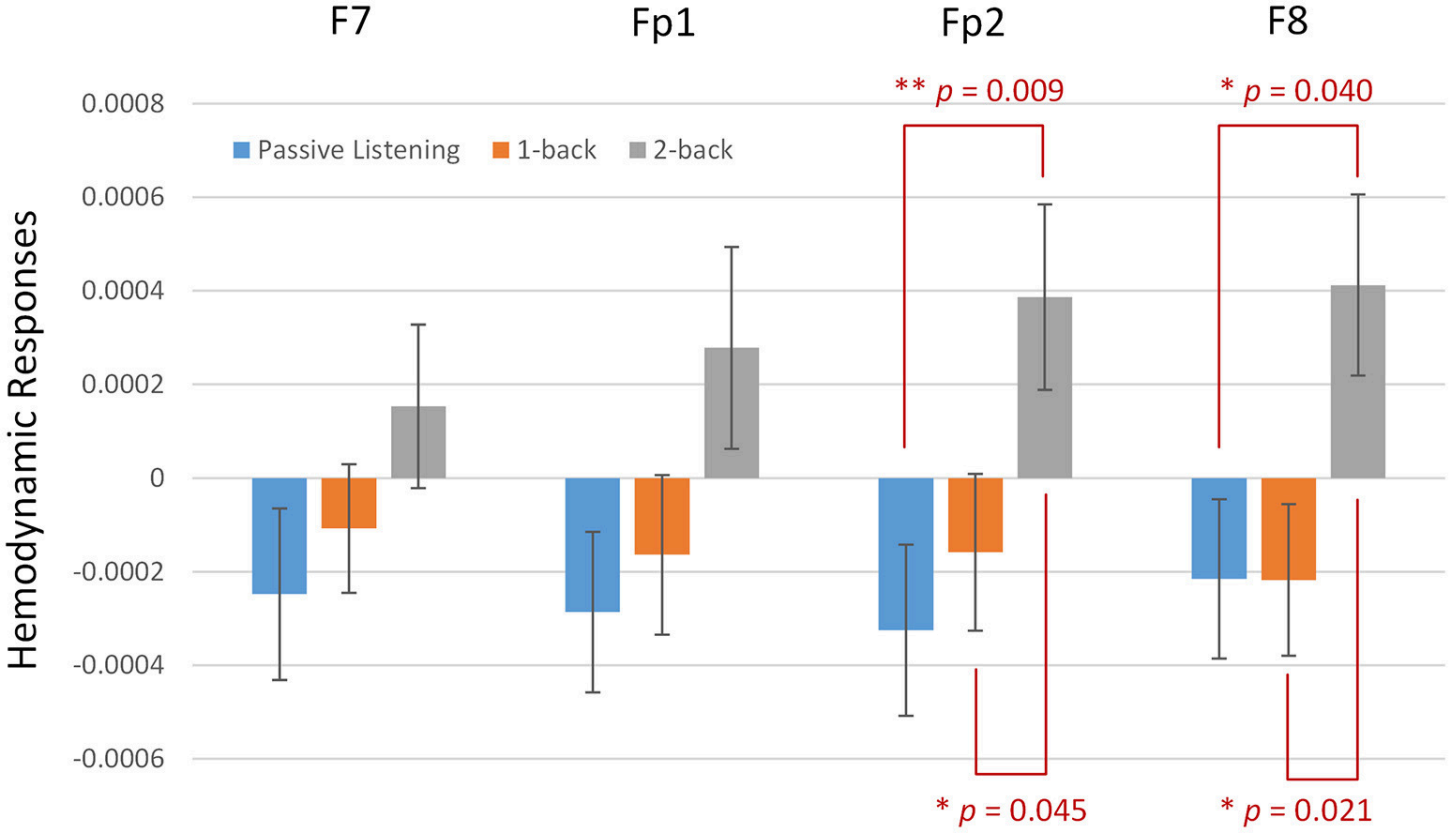
Hemodynamic responses recorded by the functional near infrared spectroscopy system when subjects were attending to the *n*-back auditory working memory tasks including three conditions: passive listening, one-back, and two-back. Stronger activations were observed from Fp2 and F8, which are localized over the right ventrolateral and orbital prefrontal cortex. Activations were more pronounced under higher working memory load. (^**^ and ^*^ denote the significant level at the 1 and 5 per cent levels, respectively).

The fNIRS signals of oxy- and deoxy-Hb were then averaged over the time points in each block and normalized according the mean value of their baseline. As illustrated in Figure 4, the hemodynamic responses recorded from the four channels of the fNIRS system were standardized and averaged across the three conditions. Stronger activations were observed from all four channels under higher auditory working memory load. The average amplitudes of oxy-Hb signals were most pronounced when the participants were attending to the two-back task. In the right VLPFC (F8), a significant difference was found between PL and 2B (*p* = 0.040), and also 1B and 2B (*p* = 0.021). The difference was also pronounced in the right OPFC (Fp2) with significance between PL and 2B (*p* = 0.009), and also 1B and 2B (*p* = 0.045). The results revealed that there are significantly stronger hemodynamic responses in the right VLPFC and OPFC when participants were attending to the auditory working memory task with high memory load.

### Correlation between anxiety scores and auditory working memory load

**Figure 5:**
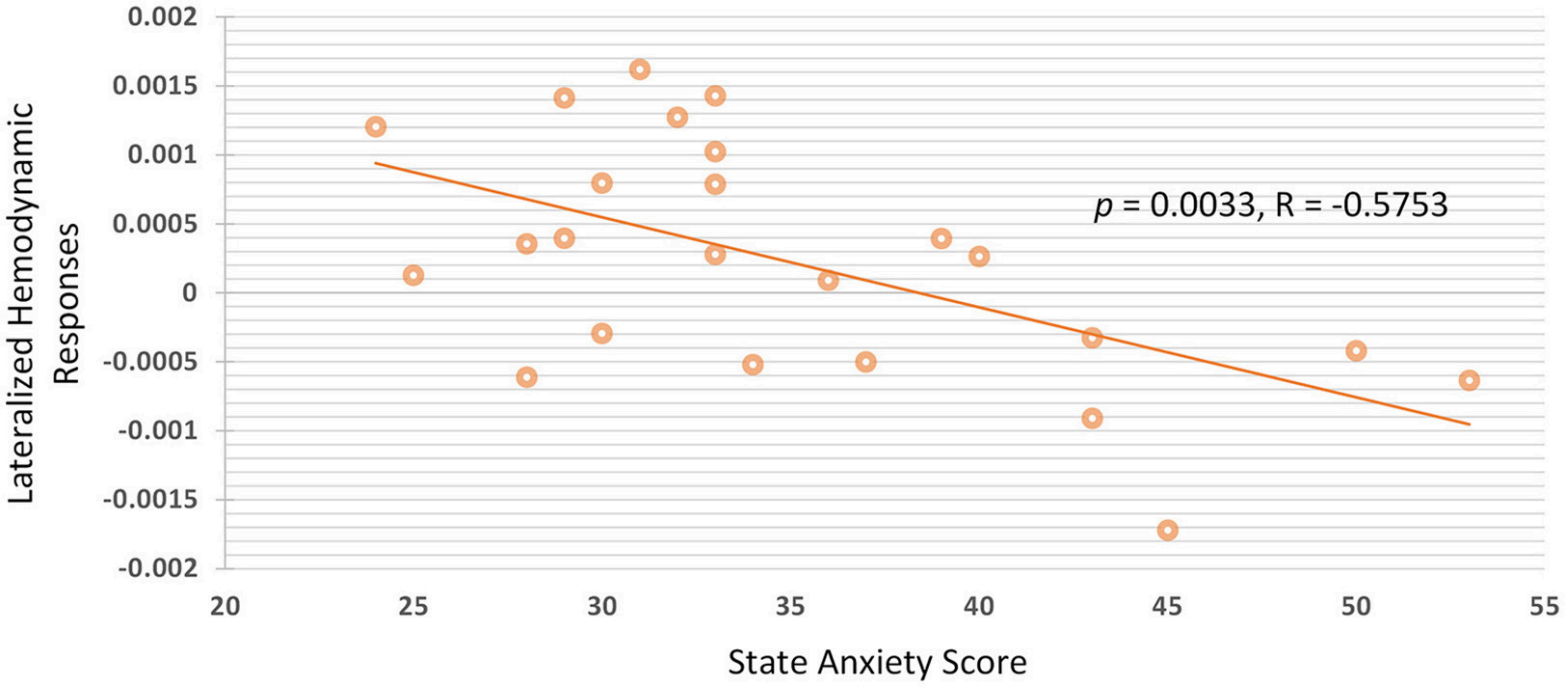
There is negative correlation between state anxiety and lateralized hemodynamic responses under higher working memory load.

Group analysis of all participants were performed by calculating the Pearson's linear correlation between anxiety scores and hemodynamic responses of the fNIRS signals. The association between state and trait anxiety with the mean oxy-Hb amplitudes and lateralized hemodynamic responses were evaluated in all of the four channels in the PFC. The significance between state anxiety and lateralized hemodynamic responses was found under higher working memory load during the 2B condition (*r* = −0.575; *p* = 0.0033), as shown in Figure 5. A negative correlation was observed between state anxiety and right lateralized hemodynamic responses, which is the activation difference between the right and left VLPFC (F7 and F8). The negative correlation between state anxiety and the lateralization index was observed to be slightly more pronounced in the female group (*r* = −0.642; *p* = 0.0243) than in the male group (*r* = −0.548; *p* = 0.0652) but did not reach the significant level. There was no pronounced correlation found between anxiety levels and the lateralization index under low working memory load conditions during 1B and passive listening conditions. We also examined the correlations between task performance and the oxy-Hb signal changes, and no significant difference was found.

In summary, increased hemodynamic responses in the right PFC were observed under higher auditory working memory load. The results also indicated that there was no obvious correlation between task performance and prefrontal hemodynamic responses of fNIRS signals. In contrast, the prefrontal right lateralization of the PFC was negative correlated with the level of state anxiety.

## Discussion

The aim of this study was to explore the effect of anxiety on auditory working memory with a portable four-channel fNIRS device. We report the following findings. First, there were increased changes in hemodynamic responses in the right orbital and ventral PFC under higher auditory working memory load during the two-back task. Second, a negative correlation was found between state anxiety and the right lateralization index of the ventral PFC. However, the correlation was not observed between trait anxiety and hemodynamic responses. This suggests that the level of fNIRS signals is correlated to the anxiety scales observed during the experiment instead of the personality of the participants. Third, the results of this study showed the feasibility of using a portable and flexible four-channel fNIRS system to evaluate the effect of anxiety on working memory performance rather than other complex neuroimaging techniques. Detailed findings and their connections with previous studies are discussed in the following subsections, with the aim of drawing a precise picture of the prefrontal hemodynamic responses underlying anxiety levels and auditory working memory.

### Increased hemodynamic reponses in the right prefrontal areas under high task load

Our results showed that there is a pronounced increment of hemodynamic responses in the right VLPFC and OPFC under high auditory working memory load. Although the increment was observed in all four channels located in the prefrontal regions, only the channels located at Fp2 and F8 showed significance. Prefrontal activation has long been reported to be correlated with working memory processes in fMRI studies (Manoach et al., 1997; D'Esposito, 2007; Barbey et al., 2013; Lara and Wallis, 2015; Riley and Constantinidis, 2016). Recently, the prefrontal region has been further emphasized as critical in auditory connections that represent key pieces of information for auditory memory recognition in primates and humans (Huang et al., 2013; Plakke et al., 2013; Plakke and Romanski, 2014; Muñoz-López et al., 2015). Consistent with the previous fMRI findings, fNIRS signals are also reportedly associated with visual working memory in adults and children (Hoshi et al., 2003; Tsujimoto et al., 2004; Schreppel et al., 2008; Sanefuji et al., 2011). Previous fNIRS studies have found that hemodynamic responses in the bilateral LPFC increased during visual *n*-back tasks (Hoshi et al., 2003; Schreppel et al., 2008), an item-recognition task (Tsujimoto et al., 2004), verbal and spatial working memory tasks (Aoki et al., 2011; Ogawa et al., 2014; Sato et al., 2014), and visual and auditory working memory tasks (Sanefuji et al., 2011). For higher visual working memory load during the *n*-back tasks using materials such as digits (Hoshi et al., 2003) or faces (Schreppel et al., 2008), previous studies have also reported the increment of the bilateral VLPFC or the anterior parts of the medial frontal cortex (Ozawa et al., 2014). Although a significant difference was only found in the right OPFC and VLPFC in this study, the increment of fNIRS signals was observed at all four channels located over the PFC (Figure 4), which is in line with previous results. Our findings were in line with those of previous studies on visual working memory. The increased hemodynamic responses in the right VLPFC and OPFC observed in the present study may reflect the involvement of retrieval processes during auditory working memory under different levels of task load.

In contrast, we observed no correlation between overall correctness and oxy-Hb activation. In previous studies, the levels of oxy-Hb activation in the VLPFC have been reported to be correlated with working memory performance. Sanefuji and colleagues demonstrated that activation in the left VLPFC of preschool children was positively correlated with the memory ability measured by the Wechsler Intelligence Scale (Sanefuji et al., 2011). Ogawa and colleagues also provided evidence to this end and proposed that there is a positive correlation between the activation of the LPFC and visual working memory performance (Ogawa et al., 2014). These previous findings suggest that there exist complicated interactions between memory load, task performance, and the prefrontal hemodynamic responses.

### Negative correlation between state anxiety and right lateralization in the PFC

Following the findings of altered hemodynamic responses during auditory working memory processes, we further identified a correlation between anxiety and the level of oxy-Hb in the PFC under high memory load. Our results showed that there is a negative correlation between state anxiety and the right lateralization index in the VLPFC. Consistent with previous findings, the correlation analysis showed that the level of negative mood, which can be measured by a profile of mood states questionnaire, is inversely associated with PFC activity during a verbal working memory task (Aoki et al., 2011, 2013; Sato et al., 2014). Furthermore, mood-cognition interaction was found in the PFC in participants of different ethnicities and language backgrounds (Sato et al., 2014). In contrast, some studies have observed a positive correlation between negative emotion and hemodynamic responses in the MPFC using a facial emotional expression of an *n*-back task, which alters the emotional states of the participants by external stimuli (Ozawa et al., 2014). The map of the relationship between mental disorders and prefrontal hemodynamic responses of fNIRS signals is still under construction, especially of mental diseases with deficits in emotional regulation. Previous studies have also reported that the lateral PFC shows reduced fNIRS signals during working memory tasks in patients with ADHD (Ehlis et al., 2008), MDD (Pu et al., 2011), and attention deficits after traumatic brain injury. Furthermore, altered prefrontal activation has also been reported after methylphenidate treatment, which is a drug used in the treatment of ADHD and depression (Ramasubbu et al., 2012). In line with these previous findings, we suggest that there are complicated interactions between emotional regulation and cognitive performance.

## Limitations

The restricted range of anxiety scores may be a limitation. Hence, we have compared the anxiety scores in this study with some previous results in different countries and culture. Two Russian studies (Knyazev et al., 2004) reported trait anxiety scores with the average of 41.3 ± 9.6 and 41.8 ± 9.9. In an US study (Blackhart et al., 2006), the trait anxiety scores were reported with the average of 40.93 ± 7.43 and 36.43 ± 6.72. In two German studies (Sehlmeyer et al., 2010; Basten et al., 2011), the trait anxiety scores were reported with the average of 33.9 ± 8.36 and 33.3 ± 5.7 and was claimed to be comparable to the values of the German normative sample with the average of 34.7 ± 8.4. In this study, trait anxiety raw scores ranged from 34 to 57 with the average score of 45.8 ± 5.8, and the raw scores of state anxiety ranged from 24 to 56 with the average score of 35.9 ± 8.6. According to these previous results, we compared the range and cultural difference of anxiety scores. First, the range of the observed anxiety scores are typically not that large in a group of healthy participants, and the standard deviations calculated are often lower than ten. The standard deviations (or ranges) of the state and trait anxiety reported in this study are comparable and consistent with those reported in the previous studies (Knyazev et al., 2004, 2016; Blackhart et al., 2006; Sehlmeyer et al., 2010; Basten et al., 2011). Second, although some previous studies have also focused on the cultural difference and anxiety level (Baloǧlu et al., 2011), they used other scores to evaluate the anxiety level rather than the STAI. In addition, the age and background may not be matched for the participants recruited with anxiety scores reported in the previous studies (Knyazev et al., 2004, 2016; Blackhart et al., 2006; Sehlmeyer et al., 2010; Basten et al., 2011). Therefore, more evidence is demanded to claim if cultural difference could be a factor to affect the conclusion, and we discussed the correlation between anxiety level and prefrontal hemodynamic responses based on the range of anxiety scores reported in this study.

The accuracy of the auditory *n*-back task is observed to be lower than the study using a similar paradigm proposed by Pallesen et al. (2010). The lower accuracy of our behavioral results may due to three reasons. First, the experimental design is slightly different from the previous study. The time for response in our study is only 1,500 ms. The shorter time for response may result in worse task performance. Second, thirty participants were recruited in this study, which were more than those recruited in the previous study (Pallesen et al., 2010). The lack of experience in music and the larger number of recruited participants may also result in lower accuracy. Third, the stimuli used in this experiment was music chords, which is more complex than traditional auditory single task. Several previous studies have discussed about the accuracy of visual and auditory *n*-back task in a larger group of participants (Jaeggi et al., 2010). For visual or auditory single task, the accuracy has been reported to be higher than 90% under both 1- and 2-back conditions. However, the correctness decreases when task complexity increased. Hence, the accuracy were observed to be lower than 90% in this study. There is often an inverse relationship between the performance and task difficulty (e.g., shorter time for reaction and increased complexity of stimuli). Although the performance may be improved by decreasing the task difficulty, the effect of anxiety may be more obvious when task difficulty is increased. The task designed in this study was intended to increase the task difficulty and observe the anxiety-induced effect on prefrontal hemodynamic responses. However, there might be a quick drop in performance after task overload. Therefore, we increased the task difficulty but set a lower and reasonable threshold to the task accuracy in order to achieve the maximum effect on hemodynamic responses induced by anxiety.

### Monitoring of brain states and cognitive evaluations using portable fNIRS solutions

By validating the high correlation between fNIRS signals and gray-matter fMRI activities in the PFC (Sato et al., 2013; Noah et al., 2015), fNIRS has been proposed as a flexible tool to observe brain hemodynamic responses for cognitive evaluations (Shimada and Hiraki, 2006; Shimada and Oki, 2012; Noah et al., 2015). Most of the previous studies have incorporated high-density fNIRS systems with up to 52 channels to record hemodynamic responses during cognitive experiments (Schreppel et al., 2008; Aoki et al., 2011, 2013; Pu et al., 2011; Sato et al., 2014), and the activation of different brain regions is identified by channels/regions of interests. Unlike the high-density and expensive fNIRS solutions, we demonstrated the possibility of using only four channels of fNIRS signals located on the scalp of the prefrontal regions to evaluate the cognitive process affected by emotion regulation. Incorporating the fNIRS devices with a lower number of channels has become a growing trend (Ehlis et al., 2008; Sanefuji et al., 2011; Ramasubbu et al., 2012; Ogawa et al., 2014; Ozawa et al., 2014), especially when conducting cognitive experiments with children as participants or when recruiting patients with mental disorders. For experiments using two- or four-channel fNIRS systems (Sanefuji et al., 2011; Ramasubbu et al., 2012), the setup time would be shorter with lower complexity of experimental preparation. In comparison with other neuroimaging modalities such as fMRI, an fNIRS system is a compact solution to observe hemodynamic responses from the prefrontal regions, and it can be more flexibly applied in vulnerable participants. To our knowledge, this study is the first to show that the working memory processes affected by emotion regulation can be observed using a four-channel portable fNIRS device. The performance of cognitive processes or mental training can therefore be quantitatively evaluated by accommodating a compact fNIRS system.

## Conclusions

We proposed an experimental paradigm of auditory working memory to evaluate the correlation between anxiety and memory load. The results revealed that there were significantly stronger hemodynamic responses in the right ventrolateral and orbital PFC when subjects were attending to the auditory working memory task with higher load. In addition, the right lateralization index of the VLPFC was negative correlated with the level of state anxiety. This study showed the flexibility of incorporating fNIRS as an index to evaluate cognitive performance. Furthermore, fNIRS can potentially be applied to functional mapping in children or patients with mental disorders (Tsujimoto et al., 2004). Since it imposes fewer constraints on behavior than fMRI, fNIRS appears to be more practical than fMRI for cognitive neuroscience investigations involving the primate cortex (Fuster et al., 2005). In addition to studies on brain functions, fNIRS may also be a useful tool for the development of brain-computer interfaces (Coyle et al., 2004; Fazli et al., 2012; Kaiser et al., 2014) or the validation of drugs for mental diseases that can cause reduction in lateral prefrontal activities accompanied by improved cognitive performance (Ramasubbu et al., 2012). In summary, we suggest that it is possible to incorporate fNIRS signals as an index of cognitive evaluation given its flexibility regarding portable applications compared to other neuroimaging techniques.

## Author contributions

Y-LT, C-FL, and S-MW conceived analytical hypothesis, performed data analysis and interpretation, and drafted, revised the work. SS, TH, and G-YL performed data analysis and interpretation. All authors approved the work for publication.

### Conflict of interest statement

The authors declare that the research was conducted in the absence of any commercial or financial relationships that could be construed as a potential conflict of interest.
